# Internal Displacement: Relationship of mental health and education of children in Swat, Pakistan

**DOI:** 10.12669/pjms.36.5.1847

**Published:** 2020

**Authors:** Nasir Ahmad, Sajjad Hussain, Nasir Shaheen

**Affiliations:** 1 Dr. Nasir Ahmad, Centre for Education and Staff Training, **University of Swat, Swat, Pakistan.**; 2 Dr. Sajjad Hussain, Centre for Education and Staff Training, **University of Swat, Swat, Pakistan.**; 3 Dr. Nasir Shaheen, Centre for Commerce and Management Sciences, **University of Swat, Swat, Pakistan.**

**Keywords:** Anxiety, Depression, Education, Internal displacement, PTSD

## Abstract

**Background and Objectives:**

Internal displacement causes mental health problems and effect education of school going children. This study intended to find the relationship between mental health problems and education of children displaced during violence in Swat Pakistan.

**Methods:**

This is quantitative co-relational study conducted in Swat, Pakistan during October 2017 to June 2018. The population of the study constitutes all the students of 25 high schools destroyed during violence. Child PTSD symptom scale, Siddiqui-Shah Depression Scale and Beck Anxiety Inventory were used to collect data from the sample (712) students.

**Results:**

High level of PTSD, depression and anxiety were found in female students and those who were not attending schools during displacement. PTSD and depression have negatively affected academic achievements of female students and those students who were out of schools irrespective of their gender.

**Conclusion:**

The study concludes that internal displacement causes mental health problems in children which can be minimize through education to a great extent.

## INTRODUCTION

Disasters whether natural or manmade, it force human beings to displace to safer places. The major cause of this internal displacement is conflicts. Dryden-Peterson^1^ found that only in 2008, 26 million people were internally displaced worldwide. Children are the most disadvantageous segment of human population as displacement makes them vulnerable to mental and physical threats. UNICEF^2^ estimated that about 17 million children were displaced within their own countries. According to Murthy^3^, in Cambodia, children were more than half of the internally displaced people although they were only 19% of the total population.

The most commonly reported psychological problems of internally displaced people were post traumatic stress disorder, depression and anxiety.^4^ Such people face problems like displacement trauma, broken social network, food, shelter, health issues and social discrimination. In recent years large number of population has been displaced. This led to increase in research activities related to mental health problems. Mujeeb et al.^5^ conducted study in Jalozai camp in Pakistan, found that internal displacement may bring psychological issues for internally displaced people. The study by Shireen et al.^6^ found a strong association between trauma and mental health. A study by Lamkaddem et al.^7^ found high prevalence of PTSD among Afghan, Iran and Somali refugees. Likewise, a study conducted in Sri Lanka by Husain et al.^8^ found that people who were displaced and resettled during short term displacement had more symptoms of mental disorders than people who had been displaced for longtime. Morina et al.^9^ studied psychiatric disorders in internally displaced persons, found highest prevalence and variation for: post-traumatic stress disorder (3–88%), depression (5–80%), and anxiety disorders (1–81%). It is concluded that internally displaced people developed high level of mental health problems. Majority of the displaced people comprised of school going children which have an effect on their routine life including education.

Education is the basic right of every child. The schools are nurseries where young minds are nurtured through education. The hindrance in maintaining education in conflict situation is self-evident as displaced children cannot afford to wait for the conflicts to end so as to exercise their right to education. In almost all of the internal displacements that took place during the last 25 years, children were deprived of equal opportunities and access to education. This deprivation of educational opportunities made these children vulnerable to mental health problems. Usually in disasters like wars schools are either destroyed or used for shelter purposes.

For the last few years a large number of people have been internally displaced in North West of Pakistan. International Displacement Monitoring Center^10^ reported that in last few years almost five million people have been internally displaced in the north-west part of Pakistan. This has been reported as the largest internal displacement in the history of Pakistan. A report by elementary and secondary education Khyber Pakhtunkhwa, Government of Pakistan 11 claimed that 175 primary schools were destroyed in Swat Valley. This destruction has made the situation worse for school going children and consequently thousands of children were left deprived of their basic right to education. A study of three districts in Khyber Pakhtunkhwa, Pakistan found evidences that around 600,000 children had missed one or more years of schooling due to conflicts.^12^ Most of the studies around the world and in Pakistan focused on mental health problems, while ignoring the relationship of mental health problems of internally displaced children with their education during displacement. This study was designed to find the relationship of internal displacement of students with mental health problems and education.

## METHODS

The research was quantitative co relational in nature. The study was conducted in the most affected northern area of Pakistan (Swat) by man-made disaster; As a result, students’ education in the region was affected. The study was approved by the ethical committee of University of Swat (Letter No. 878/QEC/UOS Dated September 25, 2017), after which the study was formally initiated. The total duration of this study was nine months started from October 2017 to June 2018.

The population of the study constituted of student studying in 13 boys and 12 girls’ high schools which were destroyed during the violence. The sample consists of 712 secondary school students who have been displaced during the violence. These include 469 male and 243 female students. The inclusion criterion for this study was secondary school students whose schools have been destroyed during the conflict. The Urdu version of Child PTSD symptom scale, Siddiqui-Shah Depression Scale and Beck Anxiety Inventory were used to collect data from the sample students. The marks of students were obtained from their respective schools. Pilot testing of the study was carried out to validate the instruments locally. The instruments were administered to 139 students randomly selected from the population of the study. The Cronbach’s coefficient (α) for Saddiqui Shah Depression scale was 0.94, Beck anxiety Inventory was 0.83 and Child PTSD symptom scale was 0.74. The instruments were administered to the sample of 712 students for data collection during the class hours. The sample children were informed that the data was being collected for research purpose and the researcher will not share their responses to their teachers and parents. Written informed consent was obtained and students were also informed that they can withdraw there written consent any time.

## RESULTS

The total respondents of this study were 712. The results (Table-I) show that 42.8% of the displaced children were living with relatives and 34.3% in rented houses. It also shows that majority of the respondents (634) were out of schools during this period. This study also found that female students developed high level of PTSD, depression and anxiety as compare to male students. One major finding of the study was that those students who were not attending school, developed high level of: PTSD, depression and anxiety as compare to those who were attending any school during displacement (Table-II). It also shows that PTSD and depression have negatively affected academic achievements of

**Table-I:** Number, Percentage and Place of living of children during Displacement.

Variable	No	Male	Female	In schools	Out of Schools
Camp	89 (12.5%)	57 (12.1%)	32 (13.2%)	2 (2.5%)	87 (13.7%)
Host Family	74 (10.4%)	49 (10.4%)	25 (10.2%)	5 (6.4%)	69(10.9%)
Relative	305 (42.8%)	204 (43.5%)	101 (41.6%)	41 (52.6%)	264(41.6%)
Rented House	244 (34.3%)	159 (33.9%)	85 (35.0%)	30 (38.5%)	214(33.8%)

Total	712 (100%)	469(100%)	243(100%)	78(100%)	634(100%)

**Table II:** PTSD, Depression and Anxiety among internally displaced children.

Variable	M	S.D	T	P
***PTSD***				
Male	37.13	11.38		
Female	45.56	12.00	9.01	0.000*
Attending school	26.87	12.86		
Not Attending	32.18	13.85	3.07	0.002*
***Depression***				
Male	44.10	17.02		
Female	64.39	11.88	13.24	0.000*
Attending school	44.25	15.45		
Not Attending	51.93	21.59	2.84	0.005*
***Anxiety***				
Male	26.51	10.60		
Female	41.80	13.75	16.00	0.000*
Attending school	26.87	12.86		
Not Attending	32.18	13.85	3.07	0.002*

- Female students.
- Those who were out of schools irrespective of their gender. Table-III.

**Table III:** Relationship of PTSD, Depression and Anxiety with academic achievements of displaced children.

***PTSD***	
Male	-0.035
Female	-0.136**
Attending school	-0.030
Not Attending	-0.139**
***Depression***	
Male	-0.028
Female	-0.217**
Attending school	0.158
Not Attending	-0.187**
***Anxiety***	
Male	-0.029
Female	-0.044
Attending school	0.189
Not Attending	-0.037

** Correlation is significant at 0.01 level.

## DISCUSSION

This study intended to find out the relationship of internal displacement with mental health and education of students displaced during violence in Swat, Pakistan. It was found that most of the displaced children were living with relatives and in rented houses in other parts of the country and most of them were not attending schools during their displacement. Internally displaced families move to such places where they have relatives or friends who can support them in such situations. Secondly they prefer places where they can afford rent of houses. These findings were supported by Ellison^13^ who observed that most of the displaced children about 85% to 90 %, were living with relatives, friends or in rented rooms in Khyber Pakhtunkhwa. The displaced children living with relative or with host families are likely to miss the opportunity of education than those children who were living in camps. These camps were established by government which were provided with some basic facilities and were also open to international humanitarian agencies. The children living in camps were more accessible to education as some educational facilities were provided to them by government and social organizations. The reports of International Displacement Monitoring Center^14^ and ICRC^15^ also confirm the finding of the study.

It was found that female children developed high level of PTSD, depression and anxiety as compare to male children. This finding is consistent with previous research findings that female developed more PTSD^16^, depression^17^ and anxiety^18^ as compare to male children. Female children developed more mental disorders irrespective of the fact that male are more exposed to trauma. On contrary research studies^19^ shows that those who are more exposed to traumatic events have more chances to develop mental disorders.

The study found that those who were not attending school developed high level of PTSD, depression and anxiety as compare to those who were attending school during displacement. Mooney et al.^20^ confirms that in conflict situation the right to education is violated and the standard development of the children is hindered. Education is critically important for displaced children because children feel normal when they go to school daily in routine just like their homes. The result of this study was in conformity with Amone-P’Olak et al.^21^, Catani et al.^22^ and Gibson et al.^23^ who found mental disorders in children who missed school in conflict zones. The provision of education is of critical importance for displaced children. The environment of school provides a sense of normality, a safe space and psychological support. During internal displacement the classroom environment can act as a safe haven in the world for a child whose life has been turned upside down by armed conflict. Fazal et al^24^ and Imran et al^25^ concludes that School is the most easily available system of care that can be used as an opportunity to treat mental health problems of children.

## CONCLUSION

Therefore, the study concludes that the internal displacement causes mental health problems in children which can be minimize through education to a great extent.

### Limitations of study

Majority of the students living in the valley were internally displaced. This research was limited to a small sample of those students whose schools have been destroyed in the time of the conflict. The use of large sample and cross sectional research design would improve the results of future studies.

### Recommendations

It was recommended that continuity of children education may be ensured during displacement and this may be incorporated in the disaster management policy of the state. In order to address mental health problems of internally displaced children, special courses that can be delivered in the form of motivational plays, music and storytelling may be incorporated in the curriculum.

### Author’s contribution

**NA:** Conception and design, responsible and accountable for the accuracy and integrity of the study.

**NA & SH:** Collection of data.

**NS & NA:** Analysis and interpretation of the data and drafting of the article.

**NA & NS:** Critical revision of the article.

**SH:** Statistical analysis.
